# Supplementary material for: Modeling glioblastoma heterogeneity as a dynamic network of cell states
Source: Mol Syst Biol. 2021 Sep 16;17(9):e10105. doi: 10.15252/msb.202010105 (PMC8444284; doi:10.15252/msb.202010105)
Supplement: Supplementary file 6 — Source Data for Figure 5 [file MSB-17-e10105-s004.zip › Figure5A_sourcedata/GSEA_3071/nosigdb_stateI.GseaPreranked.1622795145664/SUVA_NPC1_PMID_31327527.html]

Details for gene set SUVA\_NPC1\_PMID\_31327527[GSEA]

|  || Dataset | state13071 |
| Phenotype | NoPhenotypeAvailable |
| Upregulated in class | na\_neg |
| GeneSet | SUVA\_NPC1\_PMID\_31327527 |
| Enrichment Score (ES) | -0.7405042 |
| Normalized Enrichment Score (NES) | -2.9223204 |
| Nominal p-value | 0.0 |
| FDR q-value | 0.0 |
| FWER p-Value | 0.0 |
Table: GSEA Results Summary

  

Fig 1: Enrichment plot: SUVA\_NPC1\_PMID\_31327527      
 Profile of the Running ES Score & Positions of GeneSet Members on the Rank Ordered List

  

| PROBE | GENE SYMBOL | GENE\_TITLE | RANK IN GENE LIST | RANK METRIC SCORE | RUNNING ES | CORE ENRICHMENT || 1 | SERINC5 |  |  | 1066 | 0.313 | -0.2945 | No |
| 2 | GRIK2 |  |  | 1068 | 0.313 | -0.2834 | No |
| 3 | SCG3 |  |  | 2025 | -0.322 | -0.5460 | No |
| 4 | AMOTL2 |  |  | 2031 | -0.323 | -0.5356 | No |
| 5 | GLCCI1 |  |  | 2212 | -0.356 | -0.5743 | No |
| 6 | MARCKSL1 |  |  | 2215 | -0.356 | -0.5618 | No |
| 7 | BTG2 |  |  | 2318 | -0.375 | -0.5774 | No |
| 8 | ABAT |  |  | 2383 | -0.390 | -0.5815 | No |
| 9 | PCBP4 |  |  | 2480 | -0.407 | -0.5942 | No |
| 10 | TUBA1A |  |  | 2771 | -0.495 | -0.6593 | No |
| 11 | OLIG1 |  |  | 3055 | -0.638 | -0.7172 | Yes |
| 12 | CD24 |  |  | 3077 | -0.659 | -0.6991 | Yes |
| 13 | TUBB3 |  |  | 3152 | -0.731 | -0.6936 | Yes |
| 14 | STMN1 |  |  | 3171 | -0.751 | -0.6713 | Yes |
| 15 | TCF12 |  |  | 3191 | -0.769 | -0.6486 | Yes |
| 16 | PFN2 |  |  | 3252 | -0.852 | -0.6347 | Yes |
| 17 | MEST |  |  | 3307 | -0.940 | -0.6158 | Yes |
| 18 | LRRN1 |  |  | 3359 | -1.060 | -0.5916 | Yes |
| 19 | DLL3 |  |  | 3368 | -1.090 | -0.5541 | Yes |
| 20 | TAGLN3 |  |  | 3378 | -1.121 | -0.5157 | Yes |
| 21 | CHD7 |  |  | 3389 | -1.167 | -0.4759 | Yes |
| 22 | MAP2 |  |  | 3422 | -1.305 | -0.4373 | Yes |
| 23 | HES6 |  |  | 3431 | -1.366 | -0.3897 | Yes |
| 24 | SOX11 |  |  | 3469 | -1.784 | -0.3350 | Yes |
| 25 | ASCL1 |  |  | 3484 | -1.937 | -0.2682 | Yes |
| 26 | BEX1 |  |  | 3490 | -2.151 | -0.1909 | Yes |
| 27 | FXYD6 |  |  | 3503 | -2.596 | -0.0994 | Yes |
| 28 | DCX |  |  | 3505 | -2.772 | 0.0017 | Yes |
Table: GSEA details [plain text format]

  

Fig 2: SUVA\_NPC1\_PMID\_31327527: Random ES distribution      
 Gene set null distribution of ES for **SUVA\_NPC1\_PMID\_31327527**
